# SOCIAL ISOLATION AND DEPRESSIVE SYMPTOMS OF OLDER CHINESE AMERICANS: THE MEDIATION EFFECT OF ACTIVITY ENGAGEMENT

**DOI:** 10.1093/geroni/igae098.1568

**Published:** 2024-12-31

**Authors:** Ke Li, Fengyan Tang, Soonhyung Kwon, Yanping Jiang

**Affiliations:** University of New Hampshire, Durham, New Hampshire, United States; University of Pittsburgh, Wexford, Pennsylvania, United States; University of South Florida, Tampa, Florida, United States; Rutgers, The State University of New Jersey, New Brunswick, New Jersey, United States

## Abstract

Older Chinese Americans are particularly vulnerable to social isolation and its negative consequences on mental health. However, little is known about the intermediate processes through which social isolation contributes to depressive symptoms among this population. The aims of the study were (1) to examine the differential impacts of two forms of social isolation (social disconnectedness and perceived isolation) on depressive symptoms and (2) to investigate the mediating role of activity engagement. Data came from 2,075 participants in four waves of the Population Study of Chinese Elderly in Chicago Study (PINE). Social disconnectedness was measured by social network size and range, living arrangement, and marital status. Perceived isolation was measured by the perception of loneliness and lack of social support. Activity engagement was assessed by engagement with various social and cognitive activities. Results from the latent growth curve models indicated that social disconnectedness and perceived isolation were positively associated with the initial level of depressive symptoms. However, perceived isolation predicted a faster decline in depressive symptoms. The findings suggested the significant mediating effect of activity engagement. Social disconnectedness was associated with greater activity engagement, which in turn was associated with a lower initial level of depressive symptoms. Perceived isolation was associated with lower activity engagement, subsequently resulting in a higher initial level of depressive symptoms and a faster decline of depressive symptoms across waves. Intervention targeting depression among older Chinese Americans should make efforts on providing more opportunities for culturally sensitive activity engagement and social interaction and promoting social support system.

